# The Treatment, Medical Complications and Sequelæ of Typhoid Fever

**Published:** 1899-06

**Authors:** H. A. Hare

**Affiliations:** Philadelphia, Pa.; Professor of Therapeutics in the Jefferson Medical College of Philadelphia


ATLANTA

### Journal-Record of Medicine.

Successor to Atlanta Medical and Surgical Journal, Established 1855, and Southern Medical Record, Established 1870.

VOL. I. JUNE, 1899. NO. 3.

BERNARD WOLFF, M.D., DUNBAR ROY, A.B., M.D., EDITORS.

M. B. HUTCHINS, M.D., BUSINESS MANAGER.

PUBLISHED MONTHLY. OFFICE 305 AND 306 FITTEN BUILDING.

### ORIGINAL COMMUNICATIONS.

### THE TREATMENT, MEDICAL COMPLICATIONS AND SEQUELÆ OF TYPHOID FEVER.*

BY H. A. HARE, M.D.,

Professor of Therapeutics in the Jefferson Medical College of Philadelphia.

The subject which I desire to call your attention to to-day is one which is of far greater importance than it would appear at first sight, although every general practitioner, of course, recognizes that typhoid fever is a disease always with us and of sufficient severity in some cases to produce desperate illness and death. It is so large a subject that it will not be possible for me to do more than to touch upon a number of facts in connection with it, which should be carefully borne in mind.

In a paper which I read before the College of Physicians of Philadelphia thirteen months ago, I showed conclusively that there was a decrease in the frequency and in the mortality of typhoid fever

*Read at Georgia State Medical Association, Macon, April, 1899.

all over the world, which decrease is probably due to improved sanitation and a better knowledge of the methods which should be employed in the treatment of the malady.

The next point which I wish to take up deals with a question of what should be the proper treatment of the average case of typhoid fever. In the paper which I have already quoted I protested against the use of the cold plunge bath in every case of typhoid fever that came into the physicians' hands, first, on the ground that routine treatment was never rational, and second, because a certain proportion of patients present contraindications to its use, which are absolute, and a still larger class can be treated by properly modified hydrotherapy with excellent results.

In making this latter statement I am well aware that I am doing a dangerous thing, first, because a number of men, eminent in the medical profession, have given their support to the universal employment of the cold bath, and second, because there is a possibility that my own position in this matter may be misunderstood. In other words, it may be that a careless hearer will think that because I do not use the bath in every case, I do not use it in any case. This is exactly the opposite of the view which I wish to leave with you. As firmly as I believe that the plunge bath is not for every one, so do I believe that in some cases it is the best and only thing which can be employed with advantage.

I also wish to insist upon the fact that, while the disease has decreased mortality independent of such treatment, that hydrotherapy has saved a great number of lives, and if properly employed will continue to do so. The important points for the physician to decide when treating any case of disease are, first, the remedy which is needed, and second, its dose. In the opinion of those who have the largest experience, hydrotherapy is needed in every case of typhoid, and with this view I most heartily concur. When it comes to the question of dosage my point of view differs from that of some others. I believe that the mode of applying cold should be varied to the needs of the individual patient, and I have yet to see a case in which I have regretted the employment of the modified plunge bath. What, then, are the modifications which I would suggest? First, the use of cold applied to the body of the patient, who is stripped and who lies upon his bed while the nurse gives him the necessary friction and massage. The water is to be varied in its temperature according to the persistency and degree of the fever. If the patient suffers from marked hyperesthesia of the skin, so that the cold and the rubbing make him actually wretched, the first so-called spongings may be made with water at 70 deg.; later on ice-water may be employed, or if the fever is high and persistent, a piece of ice may be rubbed over the body instead of the use of water itself. In other words, the dose of cold varies with the needs of the individual. It is of vital importance that the nurse who employs this modified bath treatment should be trained to her duties. I constantly meet with nurses who conscientiously attempt to carry out my wishes without previous training, and who practically never succeed in reducing temperature of the patient more than a degree when using the plan that I have mentioned; as soon as I place a trained nurse in charge of the case, I am able, almost without an exception, to reduce the temperature many degrees.

During the past three months I have had fifty-eight cases of typhoid fever under my care; every one of these has required hydrotherapy; in only two has the persistency of the fever necessitated the use of the cold plunge, and in both of these, by a curious coincidence, and I do not consider it any more than a coincidence, hemorrhage from the bowel at once occurred. You will notice that almost without exception the temperature under the use of this form of cold falls from 2 to 4 and 6, or even 8 deg., proving two points—first, that if this form of cold is properly applied it reduces the temperature satisfactorily and produces a good reaction, and second, that if such a fall of temperature can be produced by mild means, why should we resort to the labor, the exhaustion and the excessive effect of an actual plunge? I am firmly of the belief that the active rubbing which accompanies the use of cold in the way that I have described is of great advantage to the patient, because I believe that a gentle massage given to patients suffering from typhoid fever, and who are practically taking the rest cure, is an exceedingly useful thing for the maintenance of health; second, because by these frict[illegible]ns we increase reaction, and third, as has been proved by Popischil, friction increases the loss of heat 80 per cent., and the loss of moisture through the skin, according to Weisroch, 50 per cent.

There is only one thing that can be urged against this modified form of hydrotherapy which cannot be urged against the cold plunge, and that is, that unless it is given properly it does not reduce the fever in the way in which the cold plunge necessarily reduces it, but I would ask you to remember that any remedy which is powerful for good is equally powerful for harm, and that the cold plunge cannot be used carelessly or without attention to detail any more than can the cold and friction which I have named. Whenever a physician tells me that he is unable to lower the temperature by friction and cold without the plunge, I am confident that it is because the method has not been properly employed, for only rarely is the fever so persistent as to fail to fall. Of course, in the early stages of typhoid it is a well-known fact that the fever is peculiarly resistant, not only to cold frictions but also to the plunge itself.

I next wish to call your attention to a number of variations in the character of typhoid fever, which are apt to lead to late or erroneous diagnosis. The first of these is the so-called abortive form, but in emphasizing the fact that abortive forms do occur, let me also emphasize the fact that they do so spontaneously and that it is not in our power to abort the disease. Again, let me emphasize the fact that no conscientious physician should make a diagnosis of abortive typhoid fever until every other possibility is carefully considered and until his diagnosis has been confirmed by the Widal reaction. It is an interesting fact that in most of these cases of abortive typhoid fever the onset of the disease is apt to be severe, particularly in respect to temperature which is unduly high at the start. On the other hand, typhoid fever may develop in such a mild form that all its characteristic symptoms may be masked and the patient only suffer from moderate fever and general wretchedness for several weeks. Again, exceedingly rare instances occur in which typhoid fever is present without any fever, the so-called apyretic typhoid ; here again a diagnosis should only be reached with the utmost care, and finally, let me call your attention to the fact which has so well been emphasized by Osler, that the primary effect of the typhoid infection may be exercised upon other portions of the body than the intestinal canal, and that many cases are on record in which undoubted typhoid fever has been present without there being any intestinal lesions whatever.

*Temperature Variations from the Usual in Onset.*—These variations may be due to the complicating states which are about to be described, or they are manifestations of perversions of the ordinary temperature of the initial days, occurring without assignable cause. The presence of a consolidation in the lung, or a cholecystitis, or of any serious lesion in one of the organs of the body, may entirely alter the chart in this period of the malady. In nervous children or women the irritation of the heat-centers results in a sudden rise like the more acute maladies of an infectious type. Indeed, the onset of typhoid fever in children is more apt to be ushered in by a chill and high fever than it is in adults, as has been pointed out by Jacobi and J. Lewis Smith. Thus, a case of this character is reported by Guinon (*Revue Mensuelle des Maladies de l'Enfance*, 1897, p. 236), in which a child of two and one-half years was seized with high fever and with all the symptoms of pernicious malarial infection. Nine days later it suffered from collapse with all its characteristic symptoms, and the day following passed stools which were typhoid in appearance. Collapse again occurred, and on the twelfth day symptoms of meningitis developed. Finally, a rose rash appeared, and the spleen and liver were found to be enlarged, and the case proved itself to be one of unmistakable typhoid fever. The age of the child, the sudden onset, with flushed face, the high fever, the collapse, and, finally, the meningeal symptoms, are of interest.

In some instances in which this high temperature is noted, when the physician first sees the patient, it is not in reality the earliest perversion of normal temperature, in that a mild and unnoticed fever has, perhaps, been present for some days, even though the patient has felt perfectly well. High initial temperatures indicate that the physician must be on his guard, because they mean severe infection or some grave complication which he must search for and discover, and particularly is this the case if the initial temperature is ushered in or is followed by a chill or rigor. Not very rarely careful study of the history of the patient will reveal an exposure to malarial infection, and an examination of the blood may reveal the presence of the malarial parasite.

The more sudden the appearance of the disease, and the more rapid the rise of temperature in the beginning of the first week, so much the more should one expect in general a short and even abortive attack, and, conversely, the more rapidly the temperature falls as the end of the first week is approached the better the prognosis, particularly if the daily fluctuations are marked.

Very sudden development of true hyperpyrexia, unless due to some essential complication, is very rare.

In some instances, not commonly met with, typhoid fever is ushered in by a severe chill. As already pointed out, these are most apt to appear in children, and in the majority of cases indicate the development of some coincident infection. Chills may, however, be due to the typhoid onset itself. They are met with more frequently at the onset of relapse than at the primary onset. Thus, in a case recently under the writer's care, a man of thirty-five years, after several days of malaise, was seized with a violent rigor, and became so ill at once that he was forced to go to bed, where he passed through a severe attack of the disease.

Under the name of "Sudoral typhoid fever," Jaccoud records in *La Semaine Médicale* for March 12, 1897, his belief in this special type. The onset of the malady is sudden and is accompanied by severe headache in the retro-orbital and occipital regions, with shivering, fever, and sweats, so that the patient resembles in this condition one suffering from an intermittent attack. These attacks are often quotidian, and the febrile movement is hyperpyretic. These peculiar symptoms cease by the fifth day, and are followed by the usual course of typhoid fever. Quinine does no good in these cases, and they are not due to malarial infection. A second form is characterized by the primary appearance of headache and fever and then sweating, which is profuse and asserts itself much later than in the form just described. The febrile movement is distinctly intermittent in type, but not so markedly so as in that form named. In other cases, in place of a marked rigor, the patient has a subjective sensation of coldness in some part of the body, which can also be perceived by the physician if he touches the spot. In these forms these irregular manifestations may last three weeks and then gradually cease in the fourth week. Sometimes these cases are, however, very prolonged, and Borelli has reported instances lasting seventy or ninety days. Indeed, Jaccoud regards the length of the attack as characteristic. There are practically no complications. Albuminuria is extremely rare, but intestinal hemorrhage of mild degree is not uncommon. Peritonitis from perforation, Jaccoud asserts, is quite unknown in these forms, and he regards "sudoral typhoid fever" as a mild type of the disease. Notwithstanding the close resemblance of these types to double infection by the malarial organism, and the typhoid bacillus, both Jaccoud and Borelli believe them to be pure typhoid fever, because they occur in persons who have never been exposed to malarial infection, and because quinine is useless.

The differential diagnosis is necessarily difficult in the early stages of the disease, although in general Jaccoud would have us believe that it is easy. It must depend largely upon the absence of any history of malarial exposure, upon complete development of most of the characteristic signs of typhoid fever, and, finally, upon the absence of any signs of the malarial organism in the blood and the presence of the Widal reaction.

In the well developed stage of the disease, variations from the usual temperature course are very frequently met with.

Of the cases in which the temperature is of low degree and mild we find much to say. In the first place, in very rare instances cases occur in which there is not only no fever, but actually a condition of subnormal temperature from the beginning to the end of the attack. Thus in several cases under my care, some years since, there was a characteristic temperature curve in form but not in degree, the morning temperature being distinctly subnormal, and the temperature normal, and in which the return to health consisted in a "lysis," in which the temperature gradually rose to normal instead of falling. Again, almost equally rarely there is no temperature movement whatever in the sense that the temperature is either above or below normal.

Cases of this type have been recognized for many years by close students of the disease, but are not commonly recognized by the general practitioner, who is taught in the medical schools to regard fever as a necessary symptom of the malady. Many years ago the elder Miescher recognized these cases, and Liebermeister recorded, in 1869, 139 cases of "afebrile abdominal catarrh," which he thinks were in large part due to typhoid infection, and, in 1870, 111 cases of the same character. Many of these cases showed evident enlargement of the spleen, and in some instances a roseola. Strabe (*Berliner klin. Wochmachige*, 1871, No. 30) has described 14 cases in which no fever was present, although at times the temperature was subnormal, and in which, nevertheless, the other characteristic symptoms of enteric fever were present to so marked a degree that they could not be taken for any other disease. The mortality in these cases was no less than 14.1 per cent. So, too, Fraentzel (*Zeitschrift für klinische Medicin*, 1881, p. 226) has recorded 41 cases treated in a field hospital during the Franco-Prussian war, in three of which the fever did not exceed 99.1 deg., and in the rest did not rise above 102.2 deg., and yet in which the mortality was 39 per cent. for the 41 patients. Guitéras (*Transactions of the Association of American Physicians*, 1887) records a case in which he diagnosed the condition as intestinal obstruction, in which the patient died of peritonitis, and at the autopsy the lesion of typhoid fever was found, although no fever had been present. Vallin (*Archives générale de Med.*, November, 1873; see also Liebermeister and Hagenbach, *Aus der med. klin. zu Basil*, 1869, p. 9) records a case of death due to perforation in an afebrile typhoid fever patient and another of intestinal hemorrhage in a similar case, and I have seen several cases of this character in one epidemic. In still another epidemic another instance was met with, which has been recorded in the *Memphis Lancet* for July, 1898.

In *La Province Médicale*, November 26, 1897, Weill and Piery report a case of apyretic typhoid fever, which they considered in other ways entirely typical.

Two cases of apyretic typhoid fever have also been recorded by Wendland (*Deutsche Medizinal Zeitung*, August 29, 1893). These cases were confirmed by autopsies and illustrate, at least to the satisfaction of Wendland, that temperature is not a true index of the severity of the disease.

So, too, in that condition known as "abortive typhoid fever" the severe onset and high fever may so soon be followed by moderations and signs of convalescence, with a falling temperature, that the course of the temperature may be more aberrant and the chart misleading.

Here again, however, as in all the various forms of temperature just described, the physician must not be readily led into a diagnosis of an aberrant form of typhoid fever by the knowledge that such aberrant forms occur, for these forms are so infrequent as to be curiosities, and are so rare that the probabilities in an obscure case are against their presence. Only the clear and undoubted development of a sufficient number of pathognomonic symptoms, coupled, if possible, with a positive reaction with the Widal test and with a history of recent possible typhoid infection, should cause the physician to reach a diagnosis of these types of enteric fever.

In aged persons enteric fever is usually mild in its temperature curves, and the characteristic febrile movement is so irregular and distorted as to be devoid of much diagnostic value.

In other cases the fever is peculiar in that it fails to follow the so-called normal rise in the evening and slightly lower degree in the morning, and is supplanted by a reverse type in which the morning temperature is highest.

In this connection, too, it must be remembered that in some cases (not many) during the course of the second week, the fever develops a type closely resembling that seen in remittent malarial fever. According to many writers on diseases of children, this form of the fever is by no means rare in this class of patients. Again, as this week or the third week ends, the febrile movement may even be distinctly like that of a malarial intermittent without there being any malarial infection of the patient whatever. Strümpel speaks of such cases in which distinct remittance occurred, and of others in which the fever was completely intermittent, the afternoon temperature for two or three weeks being as high as 104 deg., yet followed by morning temperature at the normal point; and Pepper has expressed the belief that these great variations are in part the result of marked sepsis and intestinal ulceration. Thus he has seen as much as seven degrees variation occur for several days in succession. Such variations should never be considered curiosities in typhoid fever, but should stimulate the medical attendant to increased endeavor to discover septic source other than the intestinal lesions, as, for example, a septic kidney. They may occur, however, in cases without complicating disease or lesions, as in one case the blood was examined repeatedly for the malarial organism with negative results, and there was no history of exposure to it. Cases of this type are also recorded by Herrington, who discusses these temperature variations in St. Bartholomew's Hospital reports for 1896. In one of these a woman of thirty-three years had severe rigors followed by high fever on the evening of the twenty-third and the morning and evening of the twenty-fourth day of the disease. These rigors were followed by a fall of fever, which amounted to a crisis, and speedy convalescence ensued. In still another case chills and fever occurred on the thirty-first, thirty-fifth and thirty-sixth days of the illness, followed by two attacks on the thirty-eighth day. These were in turn followed by crisis and recovery. In the other cases reported by Herringham a rigor occurred in one during the acme and later during lysis; in another at the onset of lysis; in another in lysis; in another a number of rigors occurred in acme and severe rigors in lysis, probably due to thrombosis.

Osler has also reported a case of this type (*Johns Hopkins Hospital Reports*, 1895, No. 5). It is well to recall the fact insisted upon by no less an authority than Janeway (*Transactions of the Association of American Physicians*, 1894) that the use of the coal-tar products in the course may have a chill-producing effect. It is well known that the external use of guaiacol will produce severe rigors.

In other cases there is present a true double infection.

There are a number of conditions which result in producing a marked and sudden fall of temperature during the periods of the fastigium and defervescence aside from the sudden drop, rarely seen, in which the fever ends by crisis instead of lysis, the patient passing into convalescence at once. The most important of these causes, both because of their degree and because of what they indicate, are hemorrhage from the bowel, or, if profuse, from any other part of the body, perforation of the bowel and the rigor preceding a complicating infection, such as pneumonia, the beginning of a relapse or because of the effect of powerful antipyretic drugs. Often great falls in temperature take place when the typhoid infection is associated with malarial infection, as already intimated.

Sometimes well-developed signs of collapse appear in the course of typhoid fever without indicating any serious accident in the course of the disease which could produce these symptoms. In this state the patient develops a rapid pulse, shallow respiration, pallor, and lividity, accompanied by a rigor or no rigor. There is usually a marked fall of temperature. Herringham (*St. Bartholomew's Reports*, 1896) asserts that these symptoms have no effect on the prognosis, and that treatment is practically unavailing.

How far constant fever occurring day after day and associated with manifestations of general loss of strength and debility can be relied upon in the diagnosis of typhoid fever is hard to determine. Certain it is that if a physician makes a diagnosis of enteric fever upon these symptoms alone, without bearing in mind the fact that similar conditions are equally well developed under other forms of infection, he will find himself in error in not a few instances. Chief among these may be mentioned tuberculosis of the lungs or peritoneum, that form of influenza in which the chief symptoms are abdominal, cases of ulcerative endocarditis, septicemia, and pyemia, and those of cholecystitis with ulceration, as in impacted gallstones. It must not be forgotten, too, that syphilitic fever may, in very susceptible persons, resemble typhoid infection. The febrile movement, rose rash, if it be scanty, malaise, and signs of general infection may readily mislead the physician.

Again in the more advanced stage (tertiary) of syphilis prolonged, low septic fever may be present.

Finally, let it not be forgotten that trichiniasis may resemble typhoid fever, for in it we have fever, pains in the limbs and back, headache, stupor, and nausea with pain in the belly and diarrhea. Points in differential diagnosis in this condition are the presence of leucocytosis (particularly losmophites) in trichiniasis and its absence in typhoid fever, puffiness of the bridge of the nose and about the eyes in trichiniasis.

Not only may the fever of these states be moderate and prolonged, and the evidences of asthenia marked, but enlargement of the spleen, diarrhea, and tympanites may be present. The difficulties in differential diagnosis in cases of suspected gall-bladder disease are increased by the fact that such disease often has its origin in an old infection of the gall-bladder due to an attack of typhoid fever months or years before, the bacillus of Eberth being present in this viscus during the entire interval, or in other cases invades the gall-bladder at the onset of the infection of the entire body, and so emphasizing the hepatic symptoms. Further than this, cases which have previously had enteric fever may also give the Widal test, although the immediate cause of the attack may be localized in the manner named. These forms of infection will be considered later on. Reference has been made to the possibility of the febrile movement resembling that of malarial fever. In some cases this form of infection is present, but in some the temperature chart is plainly that of an irregular typhoid fever.

These facts bring us face to face with a discussion of a subject about which great diversity of opinion exists, and has existed for years, namely, the question of what has been called "typho-malarial fever." At the present time it may be asserted as a fact that a separate disease entity of this character does not exist, and this is done on the basis that recent discoveries in the natural history of these diseases, particularly the recognition of the malarial germ on the one hand and the Widal test on the other, has enabled us to make an absolute diagnosis in cases in which so positive a statement has heretofore been impossible.

On the other hand, there is no doubt whatever that pure typhoid infection may result in the production of fever which closely follows the remittent and intermittent malarial types, and is often associated with so much gastric disturbance and vomiting, and so lacking in the more prominent typhoid symptoms usually seen that the picture of remittent malarial fever is clear, while the true picture of typhoid fever is clouded. Again, there can be no doubt that cases of true malarial infections occur in which the symptoms so closely resemble those of typhoid fever that a purely clinical diagnosis is almost impossible, particularly if an epidemic of typhoid fever is in full swing at the time. Finally, there can also be no doubt that it is possible for the patient to have a double infection with the bacillus of Eberth, and the plasmodium of Laveran, in which case, however, the malarial manifestations are unusually dwarfed by the typhoid poison, and only are marked at the onset of enteric fever and at its termination. To this mixed infection the term typho-malarial fever may be correctly applied to indicate not a separate disease, but a double infection. Etymologically, this term might also be used to define a condition of malarial fever in which, because of profound debility, the patient was in a typhoid state—that is, in a condition of which typhoid fever is a type. Practically, however, it should be limited in its use to the double infection just described.

As already shown, there can be no doubt that mild grades of typhoid infection take place in which the only symptoms of this disease is fever which runs a moderate course, and is accompanied by a certain degree of general debility. Often they begin rather abruptly, with a slight chill, or gradually the patient feels less and less well till he takes to his bed. These cases are characterized by well-marked remissions, it may be, and suffer from somewhat indefinite symptoms difficult of classification. They do not respond to quinine, nor do they show any typhoid symptoms, other than those named, and the diagnosis arrived at will depend largely upon whether the physician is practicing in the North or the South, is treating many cases of enteric fever or many of remittent fever, unless he is skillful with his microscope, in which case the Widal reaction for typhoid fever in a majority of cases will at some time settle the diagnosis for him, or an autopsy show typhoid lesions.

Or, on the other hand, he may find the malarial organism in the blood, which will prove that this infection is present, although it will not exclude typhoid fever, just as the Widal test will not exclude malarial infection.

Atkinson has well described the typhoid fever resembling malarial fever of the remittent type in the following words:

"From beginning to end they may develop no symptom that could not belong to this disorder, except the persistence of fever under strongly anti-malarial tereatment and the occasional occurrence of circumstances that point to a typhoidal origin. There is no intellectual cloudiness or hebetude of expression. Sleep is but slightly disturbed. The tongue remains moist and coated with a thin, whitish or yellowish fur; the appetite persists very often in some degree. There is almost never epistaxis. Constipation is commonly observed, diarrhea very rarely. There are no bloody stools, no tympanites, no iliac tenderness or gurgling. Rose spots are much more often absent than present. The patient can be restrained in bed with difficulty or under protest. Slight enlargement of the spleen may occasionally be detected, but is more frequently not observed. More severe cases, beginning more or less abruptly, develop primarily the symptoms of remittent fever, and diagnostic doubts only arise when the absolute resistance to anti-periodic treatment and the gradual appearance of typhoid symptoms excite suspicions of the incorrectness of the original diagnosis."

As Johnston has well said, "As at the present employed, the term typho-malarial fever has no determined meaning, leads to confusion and misunderstanding, is a cover for uncertainty and ignorance, and should be discouraged and abandoned."

Turning from the disturbances in the bodily temperature produced by typhoid infection, we may next study the unusual symptoms connected with the alimentary canal, and there may be no better opportunity in which to call attention to the fact that diarrhea is speedily ceasing to be a fairly constant symptom of typhoid fever. As a matter of fact, it is in a very large portion of cases supplanted by constipation from beginning to the end of the malady, although classical works nearly all regard looseness of the bowels, amounting to three or four stools a day, as the usual condition in average attacks. This is particularly the case in the typhoid fever of children in whom constipation occurs, even more commonly than in adults.

Indeed cases of this disease are recorded in which at the autopsy no signs of typhoid fever could be found in the intestines. Some of these have not been as carefully studied as they should be, but others are certainly authentic. Thus Du Cazal has recorded an instance in which the closest inspection failed to show intestinal lesions, yet typhoid bacilli, which responded to all tests, were found in the spleen, and the symptoms of the disease were present in life. The spleen, mesenteric glands, and kidneys were swollen and congested. Bacilli of typhoid were obtained not only from an abscess in the spleen, but also from vegetations in the mitral valves and from a hemorrhagic plaque on the surface of the brain. Banti and Karlinski have reported similar cases not so well proven.

Nichols and Keenan (*Montreal Medical Journal*, 1898, xxvii, p. 9) have reported nine cases of typhoid fever without intestinal lesions. So, too, Flexner and Harris (*Johns Hopkins Hospital Bulletin*, 1897, vii, p. 259) have recorded such a case, and Chiari and Kraus met with seven out of nineteen cases in five months.

Goodal (*Clinical Society's Transactions*, vol. xxx, 1897) reports two casas of enteric fever fatal during the third and fifth week respectively, in which there was no intestinal ulceration. The first patient was a boy of thirteen years, who had been ill a fortnight when admitted to the hospital; the second was a man of thirty years, who had already been ill ten days. Both of them showed all the clinical evidences of typhoid fever, and in each there was a swelling of Peyer's patches without ulceration. Similarly Fagge (*Pathological Transactions* for 1876) records a case of a man of thirty-three years, who had typhoid fever, and whose only lesion in the intestine consisted of one ill-defined purplish-red patch about the size of a shilling, situated a foot above the valve, and a little higher up, another patch with a brush surface, which was visible only when it was examined under water. So, too, in November, 1880, Moore showed before the Pathological Society of Dublin a case of enteric fever in which there was no disease of the glands of the ileum, while the spleen was extremely large, soft, and friable, and Peyer's patches were noted appearing less distinct than usual, though with no hyperemia, and did not present the shaven-beard appearance. So, too, Sydney Phillips has reported to the Clinical Society, 1891, two cases fatal after the third week, with no ulceration. Finally, Goodal points out that out of sixty-three autopsies he has held in cases of enteric fever at the Eastern Hospital, he has met with absence of ulceration in five cases; in two of these death took place early on the eighth and tenth days; in two others, as the result of some complication, on the thirty-second and seventy-third days.

Again, Hodenpyle (*British Medical Journal,* December 25, 1897) of New York has contributed a paper upon this subject, reporting a case of undoubted typhoid fever in which the intestinal lesions were absent. Brunschwig ("Is the Lesion of Peyer's Patches a Constant Symptom of Typhoid Fever?" Strasburg Thesis for 1870) has also recorded a case of this kind, and Hoeffel (*Gazette Médicale de Strasburg,* 1871, No. 14, p. 167) has done likewise, there being in his case but slight swelling and reddening of a few Peyer's patches. Schultz claimed to have met with twenty-one cases out of 300 autopsies of this disease without the characteristic ulcers in the ileum; but there is doubt as to the correctness of his statement and Mettenheimer (*Jahresberichte über die Gesammte Med.,* 1872, Bd. 2, p. 235) records an epidemic of typhoid fever occurring in the army in which in twenty-one cases the intestinal lesions were entirely limited to the colon. Banti (*La Reforma Médica,* 1887, p. 1448) and Karlinski (*Wiener Med. Wochen,* 1891, pp. 470 and 511) have also reported cases of this character.

A case is recorded in Cheadle's service at St. Mary's Hospital (*The Lancet,* July 31, 1897, p. 254) of a child of three years who died of typhoid fever and at the necropsy no ulceration was present in the intestine and Peyer's patches appeared to be normal. Beatty (*British Medical Journal,* January 16, 1897,) records two cases with a similar condition present.

Students very often seem to get the idea that the absence of diarrhea in a given case is an important point against the diagnosis of typhoid fever. On the contrary, it is so often absent that its absence is of no negative value whatever, although its presence possesses more importance. Certainly constipation is much the more frequent state as we meet the disease in Philadelphia, and as Osler well points out, diarrhea occurs in Baltimore in not more than 30 per cent. of his cases, and is an active form in only about 12 per cent. So, too, we find that in Cuschmann's clinic from 1880 to 1892 (*Deutsche Archiv. f. klin. Medicin*, 1895), diarrhea was met with in only 25 per cent. of the cases (1626 cases).

When the diarrhea is excessive, amounting to ten and twenty stools a day, the diet has been faulty in the extreme, or ulceration of the large bowel, amounting to a dysenteric state, is usually present. The character of the stools is usually, in those cases with moderate diarrhea, quite typical, but green stools in typhoid fever are occasionally met with. They have been referred to by Dreschfeld in Albutt's System of Medicine, the discoloration being seen during convalescence. Quill (*British Medical Journal*, Oct. 22, 1898, p. 1252) has recorded a case in which bright-green material was vomited on the eighth day, and later the patient passed bright-green fluid stools. There was great pain in the back. Garrod, Drysdale and Kanthack (*St. Bartholomew's Hospital Reports*, vol. xxxiii.) report three cases. The stools resembled chopped parsley, the liquid portion of the stools was filtered off and contained biliverdin, which was probably responsible for the discolored vomit.

The next question which it behooves us to consider at this time in this connection is whether diarrhea or constipation is a sign of mild and severe infection. The consensus of opinion seems to be that diarrhea is usually more active in serious cases, whether it is an instance of "purging as an effort at elimination," a favorite theory with those who are fond of using purgatives and so-called intestinal antiseptics, with the idea that by so doing they eliminate poisons and prevent their formation, or whether it is a manifestation of severe ulceration of the bowel with an associated catarrh. Ord (*Transactions of the Association of American Physicians*, 1888, vol. iii.) agrees with the view that diarrhea is usually associated with ulceration, and his opinion has been confirmed by the autopsies he has seen. Peabody states the case exactly opposite to this view. That Ord's view is not the case seems proved by the fact that advanced ulceration is often found in cases which had not had diarrhea and cases of marked diarrhea are seen in which the autopsy does not reveal much intestinal ulceration. In all probability the diarrhea is neither indicative of severe or light attacks in many cases, although if it be violent the exhaustion produced by the discharges may seriously imperil the patient's chances of recovery.

*Circulation.* The development of fever in enteric infection is accompanied by an acceleration of the pulse-rate, as it is in all such movements. With the onset of the disease the heart, not yet weakened by illness, may not only greatly quicken its beat, but also cause the pulse to be more strong than normal. As the disease progresses, however, the pulse becomes weaker and weaker in severe cases, and the heart-sounds more and more feeble, till they may be inaudible even with the most careful auscultation. With the ordinary quickening of the pulse and its ordinary alterations we have little to do at this point. The points that interest us are the unusual variations which consist chiefly in dicrotism, tachycardia, bradycardia and intermittence and relaxation of the vascular pathways on the one hand, and aberrant action of the heart as to force and sounds on the other. Dicrotism may be present for days at a time in feeble cases, and is an unfavorable sign of not great gravity unless associated with other grave symptoms. Ordinarily pulse-rates varying between 80 to 120 can be regarded by the physician with equanimity, although much depends upon the character of the pulse, and still more upon the quality of the heart sounds which should always be studied in connection with the pulse. With each ten additional beats the gravity of the condition greatly increases, and if a pulse rises to 140 or 150 per minute without some momentary exciting cause, and remains so rapid, the condition is indicative of doubtful recovery. If at the same time there is coldness of the extremities, independent of contact with ice-bags or other extraneous causes, dissolution may be imminent. Much depends, however, upon the quality of the pulse-wave. If it is full and possesses an approximate normal tension, the danger is less grave than if it is gaseous and relaxed and easily extinguished. Sometimes auscultation of the heart will show that it is acting strongly, yet pumping futilely in an attempt to fill relaxed and wide-open vessels.

It has been asserted by some clinicians that much prognostic information can be gained from the heart-sounds in typhoid fever. Thus Landouzy, Picot, Huchard, and others have formulated this conclusion, namely, that the disappearance of the first sound of the beat at the apex or at the base in the course of typhoid fever constitutes an evil sign if the pulse goes as high as 110, and that if the sound be absent and the pulse-rate becomes in excess of this number per minute, the prognosis is fatal. Of course, any condition of profound depression in the heart or general strength which can extinguish the first sound is more or less grave, but the association of this disappearance with high pulse-rate is an additional evil omen. Mongour (*La Presse Médicale,* April 21, 1897) has recently written a paper on this theme.

In still other instances the heart-sounds are like those of a fetus, the long pause being absent. This is called "embryocardia" and indicates some cardiac feebleness.

In other cases, which are rare, comparatively speaking, the pulse-rate remains at or below the normal all through the attack. This is without any particular import, and was thought by the older writers, such as Hufeland, Sauvages and Berndt, to be quite pathognomonic of this disease. Liebermeister states that a good pulse in typhoid fever rarely rises above 110.

If the circulation distinctly fails, congestion of the veins develops, but the surface of the body instead of becoming cyanotic or congested in appearance, becomes pallid and relaxed, a profuse sweat often being present, even though the temperature may be as high as 104°.

Over and above these gradual signs of circulatory failure, sudden collapse from hemorrhage or perforation may develop. A sudden diarrhea or an attack of vomiting may, however, cause a syncopal attack, and the sudden fall of high temperature associated with some complicating state may also do so. Liebermeister, though an ardent advocate of the cold bath says: "Sometimes a condition resembling a collapse is seen to follow a cold bath." So far as prognosis is concerned, care should be taken to separate the collapse of defervescence from that due to grave cardiac degeneration.

Acute endocarditis complicating typhoid fever had been reported by Carbone (*Gazette Medica di Torino,* No. 23, 1892). The patient was a young woman who had the classical symptoms and lesions of typhoid fever, and from whose endocardium were obtained typhoid bacilli. These bacilli were injected intravenously in various animals, producing the same lèsion.

Connell (*Montreal Medical Journal,* August, 1896), has also recorded a case of infectious endocarditis in typhod fever, due to the staphylococcus and involving the mitral and tricuspid valves.

